# Left ventricular myocardial function in hemodialysis patients: the effects of preload decrease in conventional, Doppler and speckle tracking echocardiography parameters

**DOI:** 10.11604/pamj.2021.38.45.9407

**Published:** 2021-01-15

**Authors:** Salma Charfeddine, Leila Abid, Rania Hammami, Amine Bahloul, Faten Triki, Samir Kammoun

**Affiliations:** 1 Department of Cardiology, Hedi Chaker University Hospital, Sfax, Tunisia

**Keywords:** Hemodialysis, left ventricular function, echocardiography, strain, speckle tracking imaging

## Abstract

**Introduction:**

our aim was to investigate the value of conventional echocardiography, pulsed Doppler and speckle tracking imaging (STI) analysis in the assessment of the left ventricular (LV) myocardial function in hemodialysis (HD) patients with preserved LV ejection fraction and to evaluate the effect of a single HD session on the LV systolic and diastolic functions.

**Methods:**

the study population consisted of 30 chronic HD patients. Echocardiography and Doppler studies were performed before and after HD. The LV global longitudinal, circumferential and radial strains were measured with two and three-dimensional STI.

**Results:**

after HD, LV dimensions, left atrium (LA) area, systolic pulmonary arterial pressure and inferior vena cava diameter decreased significantly. The peak mitral E velocity, the E/A ratio of the mitral inflow and the lateral E/E´ ratio decreased also significantly. The LV and LA volumes index and LV mass index (LVMi) decreased remarkably after HD. The 3D- LV and LA ejection fractions were unchanged after HD. Although, 3D-estimated LVEF seemed to be preserved in the HD patients, the 2D and 3D- strain rates were decreased in all directions. The global strain values improved in all directions after a single HD session. Inverse correlations were found between the LVMi, serum BNP and LV global longitudinal strain.

**Conclusion:**

in HD patients with preserved LV ejection fraction, the STI analysis may add important information concerning the subclinical LV dysfunction.

## Introduction

Chronic kidney disease (CKD) is a worldwide public health problem, which is associated with an increased morbidity and cardiovascular diseases. As compared with healthy individuals, mortality is increased by 10- to 20- fold in hemodialysis (HD) patients [1], and is especially due to ischemic heart disease and cardiomyopathy. Echocardiography is a feasible and non-invasive method to detect changes in cardiac structure and function in HD patients. The standard echocardiographic indices such as left ventricular fractional shortening (LVFS), left ventricular ejection fraction (LVEF), transmitral flow velocities are commonly used to study the cardiac status and to evaluate the LV function, but seem to be insufficient to detect LV impairment in CKD patients, who may present LV hypertrophy [2, 3]. Because of the impairment of the LV function is a major determinant of survival in HD patients [4], advanced echocardiography methods to evaluate the myocardial performance may be helpful, even if ejection fraction is still preserved. Tissue Doppler imaging (TDI) is non-invasive tool which has improved the assessment of some structural and functional properties of the myocardium [5].

TDI is pretended to be a useful diagnostic method to detect the LV dysfunction and acute changes in LV function [6, 7]. But, it has some important limitations as the angle-dependence [8]. Therefore, some new tools such as speckle tracking imaging (STI) have emerged to quantify all aspects of myocardial deformation [9]. By measuring displacement between 2 different points in a myocardial segment, strain provides a means to determine myocardial deformation, which relates to fibrosis and contractile function [9]. The average strain in the various myocardial segments yields different strain parameters as global longitudinal strain (GLS), global radial strain (GRS) and global circumferential strain (GCS). These measures are reproducible and could help to better understand the ventricular function changes linked to HD treatment. The aim of our study was to investigate the value of conventional echocardiography, pulsed TDI and speckle tracking analysis in the assessment of LV myocardial function in HD patients with preserved LV ejection fraction and to evaluate the effect of a single HD session on LV systolic and diastolic function.

## Methods

**Study population:** the study was performed in a tertiary care centre of cardiology between 01^st^ May 2014 and 28^th^ June 2014. We enrolled 30 chronic HD patients, who underwent maintenance HD treatment three times weekly. All patients were asymptomatic before inclusion. Patients with known coronary artery disease, cardiac arrhythmias, cardiomyopathy or severe valvular disease diagnosed by echocardiography were not included. All patients had preserved left ventricular ejection fraction (LVEF) of 50% or greater. All the study participants provided their consent before entering the study. After physical examination, different parameters were recorded for all patients before and after HD: height, weight, heart rate and blood pressure. Body surface area (BSA) was calculated by the Mosteller formula [10]. Blood analysis for serum electrolytes and Brain natriuretic peptide (BNP) was performed for all patients before and after HD session. Two and three - dimensional transthoracic echocardiography (TTE) and Doppler studies were also performed immediately before and after HD using a GE Vivid E9 ultrasound machine.

**Standard echocardiography:** parasternal long-axis view was used for the M-Mode measurements of left atrial diameter, interventricular septal, LV posterior wall thickness (IVST and LVPWT), LV end-diastolic and end-systolic dimensions (LVEDd and LVESd). In addition, left atrial area and volume were determined. LV fractional shortening (LVFS) was calculated as (LVEDd - LVESd)/LVEDd. LV ejection fraction (LVEFs) was calculated from LV volumes by the modified biplane Simpson rule and expressed as a percentage.

The pulsed Doppler transmitral flow velocity profile was obtained from the apical four-chamber view by the sample volume positioned below the mitral leaflets. Different parameters were evaluated: peak transmitral flow velocity in early diastole (E), peak transmitral flow velocity in late diastole (A), mitral A wave duration (dAm), E/A ratio, and the E deceleration time (DT). The isovolumetric relaxation time (IVRT) was measured with a recording simultaneous mitral filling flow and aortic ejection flow. Analysis of pulmonary venous flow was realised at the right superior pulmonary vein. The amplitude of the S (peak S), D (peak D) waves, the ratio S/D and the duration of Ap wave (dAp) were measured. The difference between the pulmonary and mitral A waves duration (dAp-Am) was determined.

Doppler tissue imaging (TDI) was performed in the four-chamber view, with the mitral and tricuspid annular planes perpendicular to the ultrasound beam. Pulsed TD sample volume was placed at the septal and lateral aspects of the mitral annulus, and at the lateral aspect of the tricuspid annulus. Measurements were made of peak systolic (S), peak early diastolic (E´), and late peak diastolic myocardial velocities (A´), and the E´/A´ratio at the lateral mitral annulus. The color M-mode Doppler flow propagation velocity (Vp) was obtained by combined color-flow Doppler and TM-mode interrogation of the mitral inflow during diastole.

**Figure 1:**
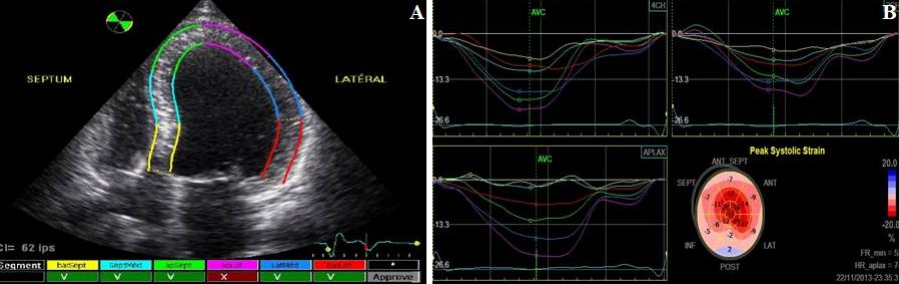
demonstration of two-dimensional speckle tracking imaging (2D-STI) analysis; after the reference points are set, the software shows the endocardial border tracking in different views; a) the apical 4-chamber view (the endocardial tracking is performed automatically through the entire cardiac cycle by 2D-STI); b) the LV longitudinal time velocity curves obtained from the apical 4- 3- and 2- chamber views in hemodialysis patients

**Speckle tracking imaging and three-dimensional echocardiography analysis:** for the tracing of the endocardium and epicardium, the following views were displayed: the apical four-chamber view, the apical two-chamber view, the apical three-chamber view and the three short-axis views, the apex of the LV, the midlevel of the LV, and the basal level of the LV. The LV endocardium and epicardium were traced automatically, and the tracings were refined with further adjustment. We performed two-dimensional strain analysis, including the global longitudinal strain (2D-GLS), radial strain (2D-GRS), and circumferential strain (2D-GCS) (Figure 1). ECG-gated subvolumes from six consecutive cardiac cycles were recorded from apical approach at end expiratory breath-hold for multi-beat reconstruction of the entire LV.

**Figure 2:**
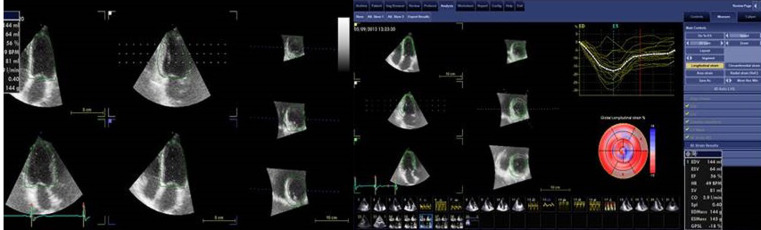
demonstration of three-dimensional speckle tracking imaging (3D-STI) analysis; after the reference points are set, the software shows the endocardial border tracking in different views; apical 4-chamber view, apical 2-chamber view, apical left ventricular longitudinal view; and three short-axis views from apex to base; the endocardial tracking is performed automatically through the entire cardiac cycle by 3D-STI

To measure LV end- diastolic and end-systolic volumes (3D-LVEDV and 3D-LVESV), LA volume, LV ejection fraction (3D-LVEF) and mass, and to perform the 3D global strain analysis, dedicated software was used (4D Auto LVQ, GE Healthcare, Horten, Norway). The 3D LV mass (LVMi) was indexed to BSA. LV hypertrophy was defined as LVMi >91 g/m^2^ [11]. At speckle tracking analysis, acceptance or rejection of LV segments were guided by the software´s recommendations. After careful assessment of the segmental strain curves by the operator, patients who had more than 3 rejected segments were excluded from the study. We defined global longitudinal (3D-GLS), radial (3D-GRS) and circumferential (3D-GCS) as the average peak systolic strain values of the 17 LV segments for the corresponding deformation parameters (Figure 2).

**Statistical analysis:** statistical analysis was performed using Statistical Package for Social Sciences version 20.0 (SPSS, Chicago, IL, USA). Continuous variables with normal distribution were presented as the mean (standard deviation, SD). Qualitative variables were given as percent. Correlations between continuous variables were assessed using Pearson´s or Spearman´s correlation analysis. To compare variables before and after HD, paired t test (for the parametric variables) and Wilcoxon test (for the non-parametric variables) were performed. A p-value under 0.05 was considered as statistically significant.

## Results

**Clinical characteristics:** the baseline demographic and clinical features of the study population are summarized in Table 1 and Table 2. The mean age was 44.93 ± 13.46 years, and 19 (63.3%) of the patients were male. The results of relevant laboratory tests before and after HD are shown in Table 2. After HD session, systolic and diastolic blood pressures, body weight, BSA, urea, creatinine and phosphorus levels decreased (all p < 0.01). BNP levels decreased also significantly after HD (253.33 (222.90) vs 221.56 (197.79) pg/mL, p < 0.01).

**Table 1:** baseline characteristics of the study population (30 patients)

Variable	Number, %
Age (years (SD))	44.93 (13.4)
Male	19 (63.3%)
Systemic hypertension	13 (43.3%)
Diabetes	4 (13.3%)
Dyslipidaemia	10 (33.3%)
Average duration of chronic HD (years (SD))	7.14 (5.9)
Fluid removal (L (SD))	2.26 (0.5)
Cause of end-stage renal disease	
Interstitial nephritis	6 (20%)
Glomerulonephritis	4 (13.3%)
Vascular nephritis	3 (10%)
Diabetic nephritis	2 (6.7%)
Hereditary	2 (6.7%)
Unknown	13 (43.3%)
HD: hemodialysis	

**Table 2:** variables before and after hemodialysis in the study population

Variable	Pre-HD	Post-HD	p-value
Clinical variables			
Systolic BP (mmHg)	126.97 (16.09)	116.93 (15.10)	**< 0.01**
Diastolic BP (mmHg)	80.23 (8.15)	73.27 (8.02)	**< 0.01**
Heart rate (bpm)	78.46 (13.68)	75.36 (10.98)	0.247
Body weight (kg)	62.31 (9.56)	60.55 (9.61)	**< 0.01**
BSA (m^2^)	1.68 (0.15)	1.65 (0.15)	**< 0.01**
Laboratory tests			
BNP (pg/mL)	253.33 (222.90)	221.56 (197.79)	**< 0.01**
Urea (mmol/L)	22.53 (5.07)	5.27 (2.99)	**< 0.01**
Creatinine (µmol/L)	849.43 (147.90)	246.17 (103.87)	**< 0.01**
Calcemia (mmol/L)	2.23 (0.20)	2.55 (0.20)	**< 0.01**
Phosphoremia(mmol/L)	1.67 (0.50)	0.66 (0.30)	**< 0.01**

Data are presented as mean (SD) BP: blood pressure, BSA: body surface area, BNP: brain natriuretic peptide

### Echocardiographic characteristics

**MM and two-dimensional echocardiographic parameters:** M-mode and two-dimensional echocardiographic changes are shown in Table 3. After HD, LV end-diastolic and end-systolic dimensions, LA area, systolic pulmonary arterial pressure (PAPS) and inferior vena cava diameter decreased significantly (Table 3). However, there was no significant difference in the LVFS and LVEFs.

**Table 3:** comparison of MM and 2D-echocardiographic parameters before and after hemodialysis in the study population

Parameter	Pre-HD	Post-HD	p-value
LA diameter (mm)	38.93 (5.28)	36.30 (4.19)	**< 0.01**
LA area (cm^2^)	19.27 (3.29)	17.00 (3.45)	**0.03**
LA volume (ml/m^2^)	38.90 (14.52)	36.74 (12.70)	**< 0.01**
LVEDd (mm)	53.20 (6.44)	48.60 (4.47)	**< 0.01**
LVESd (mm)	31.20 (4.44)	28.76 (4.38)	**< 0.01**
IVST (mm)	11.03 (2.02)	10.86 (2.06)	0.67
LVPWT (mm)	12.73 (2.34)	12.40 (2.22)	0.36
LVMi (g/m^2^)	134.37 (49.10)	111.56 (34.83)	**< 0.01**
2D-LVEDVi (mL/m^2^)	60.04 (12.89)	56.38 (11.99)	**< 0.01**
2D-LVESVi (mL/m^2^)	23.53 (6.71)	20.52 (4.70)	**< 0.01**
LVFS (%)	41.06 (4.83)	40.13 (6.67)	0.49
LVEFs (%)	61.36 (7.87)	62.36 (8.51)	0.53
Inferior vena cava diameter (mm)	13.16 (3.38)	10.23 (3.49)	**< 0.01**

Data are presented as mean (SD) LA: left atrial, IVST: interventricular septal thickness, LVPWT: left ventricular posterior wall thickness, LVEDd: left ventricular end-diastolic dimension, LVESd: left ventricular end-systolic dimension, LVMi: left ventricular mass index, 2D-LVEDVi: two-dimensional left ventricular end diastolic volume index, 2D-LVESVi: two-dimensional left ventricular end systolic volume index, LVFS: left ventricular fractional shortening, LVEFs: left ventricular ejection fraction calculated by biplane Simpson method.

**Doppler parameters:** Table 4 summarizes the measured LV Doppler findings. After HD, the peak mitral E velocity and the E/A ratio of the mitral inflow decreased significantly (p <0.01). The systolic wave velocities (S), early diastolic velocities (E´), late peak diastolic velocities (A´) at the lateral side of the mitral annulus and the E´/A´ ratio remained unchanged. Finally, the E/E´ ratio at the lateral side of the mitral annulus decreased significantly after HD (p = 0.01).

**Table 4:** comparison of Doppler indices before and after hemodialysis in the study population

Parameter	Pre-HD	Post-HD	p-value
**Mitral inflow**			
Peak E (cm/sec)	96.06 (21.86)	80.00 (21.32)	**< 0.01**
Peak A (cm/sec)	84.16 (19.87)	80.00 (20.32)	0.07
E/A ratio	1.20 (0.35)	1.04 (0.32)	**< 0.01**
DT (msec)	174.96 (41.76)	189.00 (46.85)	0.08
IVRT (msec)	89.20 (17.87)	88.13 (18.51)	0.75
**Lateral mitral annulus**			
Systolic wave (cm/sec)	10.60 (3.53)	11.43 (3.42)	0.15
E’ peak velocity (cm/sec)	12.70 (3.98)	12.00 (3.59)	0.16
A’ peak velocity (cm/sec)	11.43 (3.61)	10.66 (3.51)	0.10
E’/A’ ratio	1.23 (0.62)	1.26 (0.70)	0.63
**Venous pulmonary inflow**			
Peak S (cm/sec)	68.20 (13.64)	61.90 (15.10)	**0.03**
Peak D (cm/sec)	57.20 (10.04)	50.50 (12.40)	**< 0.01**
S/D ratio	1.21 (0.29)	1.26 (0.33)	0.51
**LV filling pressure**			
E/E’ (lateral)	8.18 (2.84)	7.12 (2.46)	**0.01**
Vp (cm/sec)	78.50 (24.76)	68.56 (23.70)	0.14
E/Vp	1.31 (0.44)	1.24 (0.44)	0.41
dAp-Am (msec)	-43.53 (35.00)	-37.30 (26.01)	0.26
PAPS (mmHg)	34.63 (8.67)	27.40 (6.74)	**< 0.01**
Elevated LV filling pressure	10 (33.3%)	1 (3.33%)	**< 0.01**

Data are presented as mean (SD)

**Three-dimensional volumetric parameters and STI analysis:** LV and LA end-diastolic and systolic volumes index and LV mass index decreased remarkably after HD. The 3D- LV and LA ejection fractions were unchanged after HD (3D- LVEF: 58.46 (7.14)% vs 58.90 (7.03)%, p = 0.74, 3D- LAEF: 37.66 (17.65) vs 39.65 (13.32), p = 0.53). Although, 3D-estimated LV ejection fraction seemed to be preserved in the hemodialysis patients, the 2D and 3D- strain rates were decreased in all directions. The global strain values improved in all directions after a single HD session (Table 5). LVEF was determined by the deformation parameters and their correlation became stronger after HD (Table 6). Inverse correlations were found between the left ventricular mass index (LVMi), two and three-dimensional global longitudinal strain (r = -0.418, p = 0.02 and r = -0.777, p < 0.01). The serum BNP levels were inversely correlated with the three-dimensional global strain rate (r = -0.673, p < 0.01). The Calcium × Phosphorus showed also inverse correlations with the global longitudinal strain (r = -0.722, p = 0.02). However, there were no correlation between impaired strain rate, hemoglobin, parathyroid hormone and blood pressure.

**Table 5:** comparison of the three-dimensional volumetric findings and the 2D and 3D- strain parameters before and after hemodialysis in the study population

Parameter	Pre-HD	Post-HD	p-value
**3D- volumetric findings**			
3D-LVEDVi (mL/m^2^)	59.67 (19.53)	54.49 (15.09)	**0.04**
3D-LVESVi (mL/m^2^)	24.57 (9.48)	21.93 (5.74)	**0.04**
3D-LAESVi (mL/m^2^)	35.60 (12.25)	31.23 (10.12)	**<0.01**
3D-LAEDVi (mL/m^2^)	22.53 (11.82)	19.06 (7.96)	**0.01**
3D-LVEF (%)	58.46 (7.14)	58.90 (7.03)	0.74
3D-LAEF (%)	37.66 (17.65)	39.65 (13.32)	0.53
LVMi (g/m^2^)	99.13 (18.06)	80.82 (18.27)	**<0.01**
**Strain parameters**			
2D-GLS (%)	-16.43 (1.73)	-18.49 (1.92)	**<0.01**
2D-GRS (%)	23.94 (9.25)	30.41 (14.10)	**0.02**
2D-GCS (%)	-20.23 (3.42)	-21.46 (4.92)	0.11
LAS (%)	31.55 (9.53)	34.77 (10.88)	**0.04**
3D-GLS (%)	-15.30 (3.23)	-17.16 (2.75)	**<0.01**
3D-GRS (%)	22.46 (6.27)	27.15 (9.10)	**0.03**
3D-GCS (%)	-19.43 (4.52)	-21.26 (3.72)	**0.04**

Data are presented as mean (SD) 3D-LVEDVi: three-dimensional left ventricular end diastolic volume index, 3D-LVESVi: three-dimensional left ventricular end systolic volume index, 3D-LAEDVi: three-dimensional left atrial end diastolic volume index, 3D-LAESVi: three-dimensional left atrial end systolic volume index, 3D-LVEF: three-dimensional left ventricular ejection fraction, 3D-LAEF: three-dimensional left atrial ejection fraction, LVMi: left ventricular mass index, 2D-GLS: two-dimensional global longitudinal strain, 2D-GRS: two-dimensional global radial strain, 2D-GCS: two-dimensional global circumferential strain, LAS: left atrial strain, 3D-GLS: three-dimensional global longitudinal strain, 3D-GRS: three-dimensional global radial strain, 3D-GCS: three-dimensional global circumferential strain.

**Table 6:** correlations between strain values, LVEF, BNP and LVMi before and after hemodialysis

	GLS		GRS		GCS		3D-GLS	
	Pre-HD	Post-HD	Pre-HD	Post-HD	Pre-HD	Post-HD	Pre-HD	Post-HD
**LVEF**	-0.421*	-0.537**	-0.410*	-0.580**	-0.261	-0.278	-0.433*	-0.619**
**LVMi**	-0.418*	-0.406*	-0.372	-0.366	-0.176	-0.216	-0.777**	-0.685**
**BNP**	-0.433*	-0.456*	-0.275	-0.331	-0.216	-0.318	-0.673**	-0.713**

* p < 0.05, **p < 0.01 LVEF: left ventricular ejection fraction, LVMi: left ventricular mass index, BNP: brain natriuretic peptide, GLS: two-dimensional global longitudinal strain, GRS: two-dimensional global regional strain, GCS: two-dimensional global circumferential strain, 3D-GLS: three-dimensional global longitudinal strain.

## Discussion

The changes in cardiac function and structure remains incompletely characterized in patients with chronic kidney disease (CKD) undergoing hemodialysis (HD). In fact, the myocardial effects of uremia and HD are difficult to determine, because micro- and macro-vascular ischemia in CKD patients significantly deteriorates myocardial mechanics and results in LV remodelling [12, 13]. Furthermore, fluid retention, volume and pressure overload, anaemia, abnormalities of calcium phosphate metabolism and hyperparathyroidism may cause cardiac dysfunction and changes in HD patients such as myocardial hypertrophy [14, 15]. Imbalance in myocardial perfusion, diastolic and systolic dysfunction can all be evident consequences of pathological hypertrophy. The relaxation impairment is usually the first sign of the deterioration of LV mechanics in the presence of increased LV mass [16]. The LV remodelling, coronary artery disease and myocardial fibrosis may also contribute to LV dysfunction in uremic patients with apparently preserved LVEF [17].

In this study, the systolic and diastolic LV functions throughout the cardiac cycle using standard TTE, Doppler, three-dimensional volumetric parameters and speckle tracking analysis were investigated before and after a single HD session in asymptomatic patients with apparently preserved LVEF. The main results of this study are: (1) the LV diastolic and systolic function were both impaired in HD patients; (2) although the global systolic LVEF estimated by conventional TTE methods seemed to be normal, longitudinal, radial and circumferential strain rates were decreased indicating that LV function was really impaired; (3) the LV volumetric parameters and function were improved after a single HD session.

Our study showed that HD induced significant changes in diastolic LV function. In several previous studies, the HD induced significant decrease in peak E of the mitral inflow and E/A ratio without any significant changes of peak A and DT [5, 18, 19]. Indeed, the conventional parameters of LV diastolic function were pre-load dependent and HD really reduced the pre-load resulting in decreased peak early filling velocities that may reveal delayed relaxation not apparent prior to HD. In our study, HD revealed delayed relaxation in 3 patients whose mitral inflow was pseudo-normal. After HD, different responses of TDI-derived diastolic velocity measurements were reported in the previous studies using TDI. In our study, after HD, there was no significant reduction in peak early diastolic (E´), late peak diastolic myocardial velocities (A´), and the E´/A´ ratio at the lateral mitral annulus. This was in accordance with some previous findings in which the TDI-derived measurements were not significantly affected after HD [7, 18, 20].

In contrast, Abid *et al*. [5], Dincer *et al*. [21] and Agmon *et al*. [22] related a significant reduction of TDI-derived indices of diastolic LV function after HD. Hung *et al*. [19] demonstrated also that TDI-derived velocities of LV function changed depending on the extent of the loading alterations. Ie *et al*. [23], in a study comparing CKD patients before and after HD, found also that volume overload before HD resulted in an underestimation of the degree of diastolic dysfunction. Hence, the LV diastolic function should be assessed in a normovolemic state, specifically at the end of the HD session. There was also a significant reduction of the E/E´ ratio after HD according to our results and some other previous data [5, 7, 19]. Therefore, these different findings suggest that TDI parameters of LV diastolic function are pre-load dependent. The disparity in these results may result in the difference of the methodology of different studies, the variation of the heart rate, the degree of change in pre-load and after-load, the clinical conditions of the studied population and the patients age. Furthermore, these previous studies have used only conventional echocardiographic indices of myocardial systolic function such as ejection fraction and fractional shortening, which are frequently found to be normal in CKD patients [24]. In fact, these methods were quite limited because of the major dependence with load-conditions variation [24]. These parameters frequently overvalue the LV function in hypertrophic cardiomyopathy [25]. In another side, the global LV function reflects the sum of all of the regional shortening in the left ventricle, and regional wall motion impairment may not reduce the LVEF unless several segments are involved [17]. So that, some studies focused on the use of TDI-derived velocities of systolic myocardial function and showed a significant increase of the peak systolic myocardial velocity (S´) reflecting the improvement of the myocardial contractility after HD [18, 24].

Nevertheless, TDI has some important limitations. Actually, if the angle of interrogation between the tissue motion and ultrasound beam is greater than 20°, the peak values will be significantly reduced. Additionally, TDI is unable to resolve the difference in motion of a myocardial segment that may be actively contracting or simply being displaced due to the tethering effect from the adjacent segment. Reverberations and side lobes can also introduce error that may overestimate or underestimate the myocardial deformation [8]. So, more recent studies focused on the regional and global strain analysis for the assessment of the LV regional function. The strain analysis is an echocardiographic method based on tracking of characteristic speckle patterns created by interference of ultrasound beams in the myocardium, and has the advantage of measuring tissue velocities and deformation in an angle-independent fashion. The technique allows for simultaneous qualification in the long and short axes and quantification of regional wall motion abnormalities in all the myocardial segments [8]. Before HD, longitudinal, radial and circumferential strains were all reduced in our patients and a single HD session resulted in an immediate decrease in the LV mass and an improvement of all volumetric parameters and analysed myocardial deformation directions. Previous data in the literature showed a similar decrease in uremic patients [8, 17, 26]. Similarly, strain analysis demonstrated early decrease in global longitudinal, radial and circumferential strain in patients with hypertrophic cardiomyopathy [25] and hypertensive cardiomyopathy [27]. Thereby, LV hypertrophy could have potentially a role in the etiology of the abnormalities identified by the strain study. In our patients, the early myocardial deformation alterations may have been caused by the LV hypertrophy as presumed from the strong correlations found between strain values and the LVMi.

There were different responses to the strain analysis after HD. As comparable to our data, Kovács *et al*. [28] and Yan *et al*. [8] reported a significant increase in all myocardial strain directions after HD. But, the strain values still altered comparing with strain values in the control groups. The improvement of myocardial deformation may be explained by the removal of overhydration gained during the interdialytic period [28]. Fagugli *et al*. [29] showed that daily HD allows optimal control of the volume fluid, blood pressure and a reduction in the LVM. Although LV hypertrophy can be a normal adaptive response to increased pressure and volume overload, it demonstrates pathophysiological changes in uremic patients, such as cardiomyocyte apoptosis, fibrosis and myocardial calcifications. Overhydration and accumulation of uremic toxins may influence the development of LV hypertrophy and LV dysfunction in CKD [8]. This suggests that hemodialysis could improve cardiac function. Our results are substantially in accordance with these findings. One of the aims of this study was to determine the ability of strain analysis to detect latent LV impairment in HD patients with apparently preserved EF. It showed that these new echocardiographic parameters could identify LV dysfunction in HD patients early in the evolution of their disease. The careful monitoring of systolic dysfunction may help in the selection of the type of appropriate renal corrective therapy to reduce the occurrence of cardiovascular disease and also to improve survival in these patients [30].

**Study limitations:** this study had several limitations. First of all, the relatively small number of patients may present a limitation for the validation of the efficiency of the methods and data. The temporal and spatial resolutions are relatively lower in the 3D-STI than in the 2D-STI. Therefore, several patients had to be excluded because the image quality in 1 or more segments was insufficient for the STI analysis. Furthermore, there was no control group to compare the different echocardiographic findings. Finally, our study was an observational study and did not include the long-term cardiovascular event rates or survival assessment of the HD patients, the relationship between the results and the cardiovascular prognosis didn´t be evaluated.

## Conclusion

In CKD asymptomatic patients with preserved LVEF, the STI analysis may add important information concerning the LV myocardial function. In fact, subclinical LV deformation and dysfunction exist in HD patients and STI offers a new tool to detect and predict the LV myocardial function changes. After a single HD treatment, the myocardial LV deformation improved in all directions. This could be due to optimal control of the volume fluid, blood pressure and a reduction in the LVM. Finally, the strain assessment may contribute to better vascular risk stratification and survival improvement in HD patients.

### What is known about this topic

- Many clinical studies in medical literature evaluated the effects of hemodialysis on left ventricular myocardial systolic and diastolic functions;
- The echocardiographic findings remained controversial in the assessment of myocardial function, and identifying early subclinical changes in hemodialysis patients;
- The speckle tracking echocardiography is a relatively new non-invasive imaging technique that allows an objective assessment of the regional and global myocardial function.

### What this study adds

- The present study highlights the importance of the echocardiographic study to detect progressive changes in the hemodialysis patients before and after a single hemodialysis session;
- The conventional echocardiography can detect some differences in hemodialysis patients but it is incapable to reveal differences in intrinsic myocardial functions: thereby, the evaluation of speckle tracking echocardiography can be useful for identifying previously undetected differences and subclinical changes before and after a single hemodialysis session. The present study showed the 2D and 3D- strain rates were decreased in all directions in the study population; the global strain values improved in all directions after hemodialysis; inverse correlations were found between the left ventricular mass index, serum BNP and left ventricular global longitudinal strain;
- Considering our results and the large heterogeneity of echocardiographic pattern generally found in hemodialysis, several possible clinical applications of deformation parameters approach can be advised.

## References

[1] Foley RN, Parfrey PS, Sarnak MJ (1998). Epidemiology of cardiovascular disease in chronic renal disease. J Am Soc Nephrol.

[2] Salvetti M, Muiesan ML, Paini A, Monteduro C, Bonzi B, Galbassini G (2007). Myocardial ultrasound tissue characterization in patients with chronic renal failure. J Am Soc Nephrol.

[3] Foley RN (2003). Clinical epidemiology of cardiac disease in dialysis patients: left ventricular hypertrophy, ischemic heart disease, and cardiac failure. Semin Dial.

[4] Bansal N, Keane M, Delafontaine P, Dries D, Foster E, Gadegbeku CA (2013). A longitudinal study of left ventricular function and structure from CKD to ESRD: the CRIC study. Clin J Am Soc Nephrol.

[5] Abid L, Rekik H, Jarraya F, Kharrat I, Hachicha J, Kammoun S (2014). Acute hemodialysis effects on doppler echocardiographic indices. Saudi J Kidney Dis Transpl.

[6] Yalçin F, Kaftan A, Muderrisoğlu H, Korkmaz ME, Flachskampf F, Garcia M (2002). Is Doppler tissue velocity during early left ventricular filling preload independent?. Heart.

[7] Graham RJ, Gelman JS, Donelan L, Mottram PM, Peverill RE (2003). Effect of preload reduction by haemodialysis on new indices of diastolic function. Clin Sci (Lond).

[8] Yan P, Li H, Hao C, Shi H, Gu Y, Huang G (2011). 2D-speckle tracking echocardiography contributes to early identification of impaired left ventricular myocardial function in patients with chronic kidney disease. Nephron Clin Pract.

[9] Burns AT, La Gerche A, D´hooge J, MacIsaac AI, Prior DL (2010). Left ventricular strain and strain rate: characterization of the effect of load in human subjects. Eur J Echocardiogr.

[10] Mosteller RD (1987). Simplified calculation of body-surface area. N Engl J Med.

[11] Olivotto I, Maron MS, Autore C, Lesser JR, Rega L, Casolo G (2008). Assessment and significance of left ventricular mass by cardiovascular magnetic resonance in hypertrophic cardiomyopathy. J Am Coll Cardiol.

[12] Thorstensen A, Dalen H, Hala P, Kiss G, D´hooge J, Torp H (2013). Three-dimensional echocardiography in the evaluation of global and regional function in patients with recent myocardial infarction: a comparison with magnetic resonance imaging. Echocardiography.

[13] Hayat D, Kloeckner M, Nahum J, Ecochard-Dugelay E, Dubois-Randé J-L, Jean-François D (2012). Comparison of real-time three-dimensional speckle tracking to magnetic resonance imaging in patients with coronary heart disease. Am J Cardiol.

[14] Schärer K, Schmidt KG, Soergel M (1999). Cardiac function and structure in patients with chronic renal failure. Pediatr Nephrol.

[15] London G (2001). Pathophysiology of cardiovascular damage in the early renal population. Nephrol Dial Transplant.

[16] Kovács A, Apor A, Nagy A, Vágó H, Tóth A, Nagy AI (2014). Left ventricular untwisting in athlete´s heart: key role in early diastolic filling?. Int J Sports Med.

[17] Chen R, Wu X, Shen L-J, Wang B, Ma M-M, Yang Y (2014). Left ventricular myocardial function in hemodialysis and nondialysis uremia patients: a three-dimensional speckle-tracking echocardiography study. PLoS One.

[18] Ouali S, Abroug S, Neffeti E, Taamalah S, Hammas S, Ben Khalfallah A (2010). [Effects of acute decrease in preload on echocardiographic indices of systolic and diastolic function of the left ventricle in children with end-stage renal disease (ESRD), Comparative study before and after hemodialysis]. Ann Cardiol Angeiol (Paris).

[19] Hung K-C, Huang H-L, Chu C-M, Chen C-C, Hsieh I-C, Chang S-T (2004). Evaluating preload dependence of a novel Doppler application in assessment of left ventricular diastolic function during hemodialysis. Am J Kidney Dis.

[20] Bauer F, Jamal F, Douillet R, Le Roi F, Bouchoule I, Bizet-Nafeh C (2001). [Acute changes in load: effects of myocardial velocities measured by doppler tissue imaging]. Arch Mal Coeur Vaiss.

[21] Dincer I, Kumbasar D, Nergisoglu G, Atmaca Y, Kutlay S, Akyurek O (2002). Assessment of left ventricular diastolic function with Doppler tissue imaging: effects of preload and place of measurements. Int J Cardiovasc Imaging.

[22] Agmon Y, Oh JK, McCarthy JT, Khandheria BK, Bailey KR, Seward JB (2000). Effect of volume reduction on mitral annular diastolic velocities in hemodialysis patients. Am J Cardiol.

[23] Ie EHY, Vletter WB, ten Cate FJ, Nette RW, Weimar W, Roelandt JRTC (2003). Preload dependence of new Doppler techniques limits their utility for left ventricular diastolic function assessment in hemodialysis patients. J Am Soc Nephrol.

[24] Hayashi SY, Brodin L-A, Alvestrand A, Lind B, Stenvinkel P, Mazza do Nascimento M (2004). Improvement of cardiac function after haemodialysis, Quantitative evaluation by colour tissue velocity imaging. Nephrol Dial Transplant.

[25] Popović ZB, Kwon DH, Mishra M, Buakhamsri A, Greenberg NL, Thamilarasan M (2008). Association between regional ventricular function and myocardial fibrosis in hypertrophic cardiomyopathy assessed by speckle tracking echocardiography and delayed hyperenhancement magnetic resonance imaging. J Am Soc Echocardiogr.

[26] Wang H, Liu J, Yao X, Li J, Yang Y, Cao T (2012). Multidirectional myocardial systolic function in hemodialysis patients with preserved left ventricular ejection fraction and different left ventricular geometry. Nephrol Dial Transplant.

[27] Galderisi M, Esposito R, Schiano-Lomoriello V, Santoro A, Ippolito R, Schiattarella P (2012). Correlates of global area strain in native hypertensive patients: a three-dimensional speckle-tracking echocardiography study. Eur Heart J Cardiovasc Imaging.

[28] Kovács A, Tapolyai M, Celeng C, Gara E, Faludi M, Berta K (2014). Impact of hemodialysis, left ventricular mass and FGF-23 on myocardial mechanics in end-stage renal disease: a three-dimensional speckle tracking study. Int J Cardiovasc Imaging.

[29] Fagugli RM, Reboldi G, Quintaliani G, Pasini P, Ciao G, Cicconi B (2001). Short daily hemodialysis: blood pressure control and left ventricular mass reduction in hypertensive hemodialysis patients. Am J Kidney Dis.

[30] de Mattos AM, Siedlecki A, Gaston RS, Perry GJ, Julian BA, Kew CE (2008). Systolic dysfunction portends increased mortality among those waiting for renal transplant. J Am Soc Nephrol.

